# The prognostic role of the immunohistochemical expression of S100 in meningiomas

**DOI:** 10.1007/s00432-022-04186-9

**Published:** 2022-07-15

**Authors:** Felix Behling, Christina Fodi, Marco Skardelly, Frank Paulsen, Ghazaleh Tabatabai, Jürgen Honegger, Marcos Tatagiba, Jens Schittenhelm

**Affiliations:** 1 Center for Neuro-Oncology, Comprehensive Cancer Center Tübingen-Stuttgart, University Hospital Tübingen, Eberhard-Karls-University Tübingen, Tübingen, Germany; 2 Department of Neurosurgery, University Hospital Tübingen, Eberhard-Karls-University Tübingen, Hoppe-Seyler Street 3, Tübingen, Germany; 3 Hertie Institute for Clinical Brain Research, Tübingen, Germany; 4 Department of Radiation Oncology, University Hospital Tübingen, Eberhard-Karls-University Tübingen, Tübingen, Germany; 5 Department of Neurology and Interdisciplinary Neuro-Oncology, University Hospital Tübingen, Eberhard-Karls-University Tübingen, Tübingen, Germany; 6 German Cancer Consortium (DKTK), DKFZ Partner Site Tübingen, Tübingen, Germany; 7 Cluster of Excellence (EXC 2180) “Image Guided and Functionally Instructed Tumor Therapies”, Eberhard-Karls University Tübingen, Tübingen, Germany; 8 Department of Neuropathology, University Hospital Tübingen, Eberhard-Karls-University Tübingen, Tübingen, Germany

**Keywords:** Meningioma, S100, Prognosis, Recurrence-free survival, Tissue microarray

## Abstract

**Background:**

Despite best clinical management, meningioma patients experience tumor recurrence. Efforts have been made to improve the prognostic stratification of meningiomas. Recently, a multi-faceted molecular classification suggested that the marker S100 is associated with a favorable outcome, making a further analysis in a larger cohort interesting.

**Materials and methods:**

The immunohistochemical staining for S100 was analyzed in 1669 paraffin-embedded meningioma samples. The distribution and association with clinical data and progression-free survival via radiographic tumor recurrence were assessed.

**Results:**

Of 1669 cases, 218 tumors showed strong S100 expression (13.1%). A significantly higher frequency of S100 positive meningiomas was observed in meningiomas of female patients, tumors with spinal and convexity/falx location, primary tumor surgery, NF2, higher extent of resection, lower WHO CNS grade, adjuvant radiotherapy and recurrence-free tumors during follow-up. Univariate analysis revealed a favorable progression-free survival for meningiomas with S100 expression (*p* = 0.0059) but not in the multivariate analysis. Higher S100 frequency was independently associated with female gender (*p* = 0.0003), NF2 (*p* < 0.0001), tumor location (*p* < 0.0001) and lower WHO CNS grade (*p* = 0.0133).

**Conclusions:**

The positive prognostic impact of S100 is mostly attributed to the confounding clinical factors gender, tumor location, NF2 status and WHO CNS grade.

## Introduction

Meningiomas are extra-axial tumors originating from the arachnoid cap cells of the meninges (Louis et al. 2021). With an incidence of 9.12/100,000 per year, they represent the most common primary intracranial tumor (Ostrom et al. 2021). Meningiomas are usually benign slow growing tumors and patients can be cured by microsurgical resection. Furthermore, radiotherapy can be applied as primary treatment in selected cases (Goldbrunner et al. 2021). Based on the histopathological grading, approximately 20% of meningiomas show more aggressive or even malignant characteristics, resulting in a higher rate of tumor recurrence (Louis et al. 2021). Since there are no established treatment options for meningioma patients besides surgical excision and radiotherapy, it is important to identify patients with a higher risk of tumor recurrence to adjust the follow-up management accordingly. In addition to long established prognostic factors such as the histopathological WHO classification (Louis et al. 2021), the extent of tumor resection (Behling et al. 2021a; Simpson 1957) or molecular drivers (TERT promotor mutation and homozygous CDKN2A deletion) (Sievers et al. 2020; Spiegl-Kreinecker et al. 2018), several aspects that influence the progression free survival where identified over the last years (Behling et al. 2020, 2021b; Sahm et al. 2016; Sievers et al. 2020). Recently, an integrative molecular classification system for meningiomas has been described. Based on DNA somatic copy-number aberrations, DNA somatic point mutations, DNA methylation and mRNA abundance, four prognostically distinct molecular groups were defined. The subgroup with the best progression-free survival showed a distinctly higher rate of S100B levels in the proteome-analysis (Nassiri et al. 2021). The calcium-binding, low-molecular weight protein S100B is mainly found in astrocytes (Donato 1999) of the central nervous system and is otherwise used in neuropathological diagnostics to assess neural crest origin in tumors such as schwannomas, neurofibromas and malignant nerve sheath tumors (Louis et al. 2021). Except for 1 study with 63 samples reporting a higher frequency of S100B expression in benign meningiomas (Hancq et al. 2004), there is little information regarding the prognostic role of S100 in meningiomas. We, therefore, analyzed the frequency and prognostic value of the immunohistochemical expression of S100 protein in a large retrospective meningioma cohort.

## Materials and methods

### Study cohort and clinical data collection

Patients that were surgically treated for a meningioma in our institution between July 2003 and March 2017 were considered for this retrospective analysis. The following clinical data were collected by reviewing electronic treatment records and imaging data: age at diagnosis, gender, tumor status (primary or recurrent meningioma), neurofibromatosis type 2 (NF2), tumor localization, WHO grade, extent of resection [according to Simpson (Simpson 1957)], adjuvant radiotherapy. All patients that were treated for meningioma in the mentioned time period were considered for inclusion (*n* = 2168). Cases were excluded if no patient consent was available (*n* = 156), clinical records were incomplete (*n* = 184), tumor tissue was unavailable or immunohistochemical staining inconclusive (*n* = 159). Finally, 1669 meningiomas were included in the analysis.

### Construction of tissue microarrays and immunohistochemical staining for S100

**Fig. 1:**
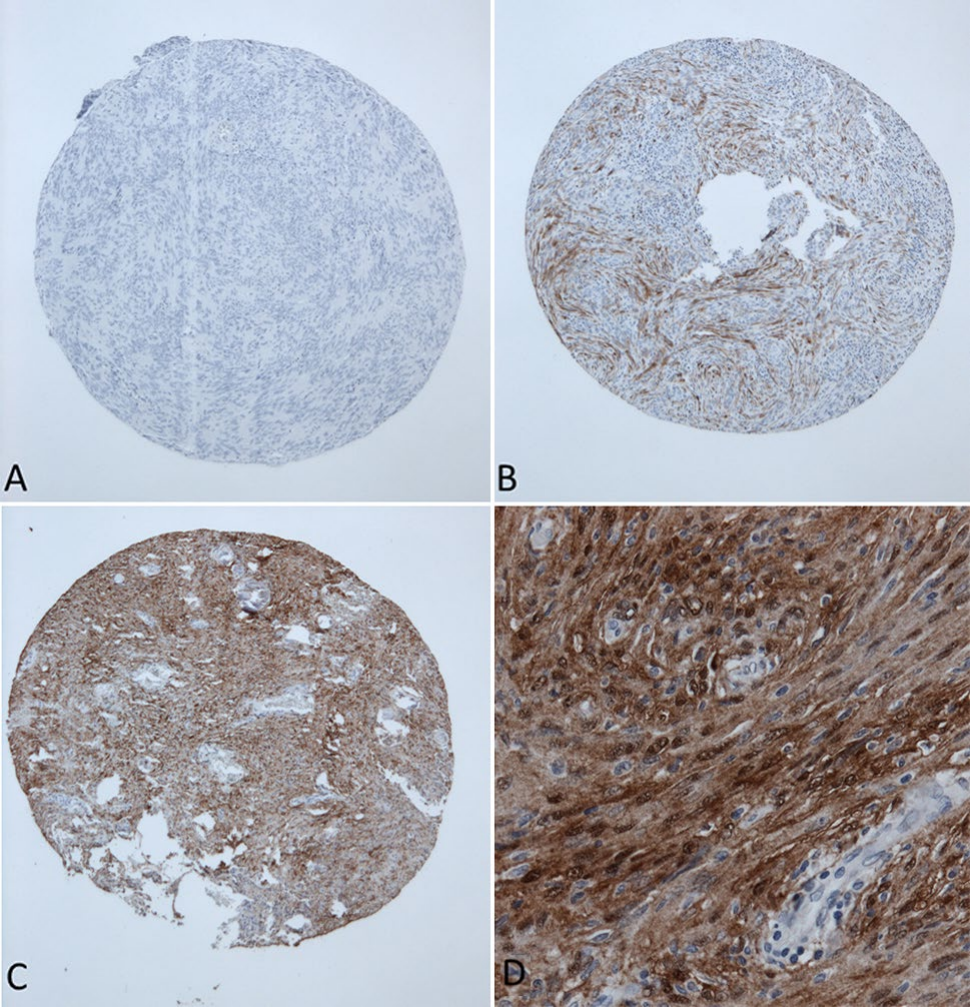
Representative S100 immunohistochemistry stains of 1000 µm sized tissue microarray punches. **A** Score 0, **B** score 1, **C** score 2, **D** enlargement showing cytoplasmatic and nuclear staining of S-100 in tumor cells (200× magnification)

Formalin-fixated and paraffin-embedded (FFPE) tumor tissue samples from the archive of the Department of Neuropathology were used to construct tissue microarrays (TMA). Hematoxylin and eosin stains were reviewed, and representative tumor areas identified. Two sample cylinders measuring 1 mm in diameter were extracted from FFPE tumor samples and aligned in recipient paraffin blocks in a checker-board pattern. A conventional tissue microarrayer was used (Beecher Instruments, Sun Prairie, Wisconsin, USA). TMA blocks were cut with a microtome, producing 4 μm slices that were dried at 80° for 15 min. Immunohistochemical staining for S100 (polyclonal rabbit anti S-100 antibody, Z0311 1:4000, Dako, Glostrup, DK) was done with a Ventana BenchMark immunostainer (Ventana Medical Systems, Tucson, Arizona, USA). Epitope unmasking pretreatment was performed as Heat Induced Epitope Retrieval with OptiView Cell conditioning solution (CC1 8 min, Ventana). Primary antibody incubation was done for 8 min at 42 °C at 1:4000 dilution. Antigen–antibody reaction was visualized using the Ventana OptiView Universal DAB Detection kit (OptiView Linker 8 min, HRP Multimer 8 min, H202/DAB 8 min, Copper 4 min). Diaminobenzidine served as brown chromogen. This antibody reacts strongly with human S100B, and only very weakly with S100A and is commonly used in neuropathology practice. Tumors were evaluated for cytoplasmatic and nuclear staining for S100. After initial screening of S100 stained TMA slides, only two staining patterns were noticed in tumor samples. Therefore, a reproducible three-tiered immunohistochemical score was applied (score 0: samples with less than 1% positive tumor cells or fully absent staining, score 1: heterogenous expression or focally positive up to 75% positive tumor cells and score 2: showing diffuse homogenously cytoplasmatic staining for S100 of at least 75% of tumor cells in at least one of the two tumor samples, Fig. 1A–C). After preliminary statistical evaluation, the three-tiered score did not reveal prognostic differences. The score was then dichotomized and samples with a score of 2 (> 75% immunopositivity) were regarded as immunopositive. Cerebral and cerebellar cortex were as used as S100-specific positive controls. The epithelial cells from the breast cancer metastasis sample were used as negative control. Furthermore, endothelial cells within meningioma tissue served as additional internal negative controls (for example see Fig. 1D). The immunohistochemical assessment was done by two independent researchers. For cases with a differing S100 result, a consensus was found.

### Statistical analysis

The Chi-squared test according to Pearson and a univariate Cox proportional hazard analysis were applied for contingency analyses of S100 expression and clinical factors. A binary logistic regression was done for the assessment of factors associated with S100 expression. Kaplan–Meier analysis was done for univariate prognostic assessment using the log-rank test. The multivariate analysis was done applying the cox proportional hazard analysis and the Wald test. A significance level of *α* < 0.05 was applied. For the definition of a prognostic cutoff for patient age at diagnosis a classification and regression tree (CART) analysis was done. The interrater agreement was expressed by Cohen’s Kappa. The software JMP® Statistical Discovery Software, version 16.0.0 (Cary, NC: SAS Institute Inc.; 1989) was used for the statistical analysis.

## Results

### Distribution of S100 immunopositivity

After the immunohistochemical assessment by two researchers, a mismatch result was documented in 43 cases for which a consensus was found. The interrater consensus was 97.4% (1626/1669). Cohen’s Kappa was 0.88 (almost perfect agreement).

Among 1669 meningioma tissue samples, strong S100 immunopositivity was observed in 218 cases (13.1%). Staining was mainly cytoplasmic but additional nuclear S100 positivity was frequently observed in tumor cells with cytoplasmic staining (Fig. 1D). Lymphocytes and tumor vessels were negative for S100. There was a significant difference with a higher rate of S100 positive meningiomas in female patients (15.0% compared to 8.3%, *p* = 0.0003). There was no staining difference regarding the CART-specific cut off at 41.5 years. Regarding tumor location, spinal meningiomas showed the highest rate of S100 positivity (23.8%), followed by convexity/falx (18.2%) and the lowest rate for skull base meningiomas (7.1%, *p* < 0.0001). The frequency of S100 immunopositivity was approximately twice as high in primary meningiomas compared to recurrent meningiomas (13.8% vs. 7.6%, *p* = 0.0147). The rate of S100 positive cases was almost 3 times as high in meningiomas from patients suffering from NF2 (11.0% vs. 4.1%, *p* < 0.0001). With higher CNS WHO grading, the frequency of S100 immunopositivity decreased (CNS WHO grade 1: 14.2%, grade 2: 9.3%, grade 3: 0%, *p* = 0.109). Among the 15 different histological subtypes, marked differences were observed (*p* < 0.0001). Especially high S100-positive rates were observed for transitional (22.5%), fibroblastic (24.8%) and psammomatous meningiomas (36.2%). There was no significant difference in S100 status regarding MIB1 immunopositivity. S100 expression was less frequent in cases that experienced tumor recurrence after meningiomas resection (7.3% vs. 14.3%, *p* = 0.0011). The distribution of S100 immunopositivity is displayed in Table 1.

**Table 1:** Distribution of S100 expression according to clinical and histopathological characteristics (Pearson’s Chi-squared test)

	*N* (%)	S100 expression (N, %)		*p* value
		Positive	Negative	
Gender				
Female	1197 (71.7)	179 (15.0)	1018 (85.1)	0.0003*
Male	472 (28.3)	39 (8.3)	433 (91.7)	
Age				
≥41.5	1456 (87.2)	184 (12.6)	1272 (87.4)	0.1786
< 41.5	213 (12.8)	34 (16.0)	179 (84.0)	
Location				
Skull base	850 (50.9)	60 (7.1)	790 (92.9)	< 0.0001*
Convexity/falx	659 (39.5)	120 (18.2)	539 (81.8)	
Spinal	160 (9.6)	38 (23.8)	122 (76.3)	
Prim/Rec				
Primary	1471 (88.1)	203 (13.8)	1268 (86.2)	0.0147*
Recurrent	198 (11.9)	15 (7.6)	183 (92.4)	
NF2				
Yes	218 (13.1)	24 (11.0)	194 (89.0)	< 0.0001*
No	1451 (86.9)	59 (4.1)	1392 (95.9)	
Simpson grade				
I/II/III	1159 (70.9)	173 (14.9)	986 (85.1)	0.0006*
IV/V	475 (29.1)	41 (8.6)	434 (91.4)	
CNS WHO grading				
1	1323 (79.3)	188 (14.2)	1135 (85.8)	0.0109*
2	323 (19.4)	30 (9.3)	293 (90.7)	
3	23 (1.4)	0 (-)	23 (100.0)	
Histology				
1				
Angiomatous	34 (2.0)	0 (-)	34 (100.0)	< 0.0001*
Fibroblastic	125 (7.5)	31 (24.8)	94 (75.2)	
Lymphocyte rich	1 (0.1)	0 (–)	1 (100.0)	
Meningothelial	823 (49.3)	92 (11.2)	731 (88.8)	
Metaplastic	20 (1.2)	4 (20.0)	16 (80.0)	
Microcystic	32 (1.9)	2 (6.3)	30 (93.8)	
Psammomatous	58 (3.5)	21 (36.2)	37 (63.8)	
Secretory	46 (2.8)	0 (–)	46 (100.0)	
Transitional	169 (10.1)	38 (22.5)	131 (77.5)	
NOS	16 (1.0)	0 (–)	16 (100.0)	
2				
Atypical	290 (17.4)	30 (10.3)	260 (89.7)	
Chordoid	32 (1.9)	0 (–)	32 (100.0)	
Clear Cell	0 (–)	0 (–)	0 (–)	
3				
Anaplastic	17 (1.0)	0 (–)	17 (100.0)	
Papillary	0 (–)	0 (–)	0 (–)	
Rhabdoid	6 (0.4)	0 (–)	6 (100.0)	
MIB1				
< = 4.6% or missing	1362 (85.5)	183 (13.4)	1179 (86.6)	0.2756
> 4.6%	231 (14.5)	25 (10.8)	206 (89.2)	
Tumor recurrence				
Yes	314 (21.4)	23 (7.3)	291 (92.7)	0.0011*
No	1155 (78.6)	165 (14.3)	990 (85.7)	
Adjuvant RT				
Yes	71 (4.8)	1 (1.4)	70 (98.6)	0.0032*
No	1401 (95.2)	188 (13.4)	1213 (86.6)	

Asterisks (*) mark statistically significant results, *CNS* central nervous system, *MIB1* proliferation marker, *NOS* not otherwise specified, *NF2* neurofibromatosis type 2, *WHO* World Health Organization, *RT* radiotherapy

### Binary logistic regression of factors associated with S100 expression

The multivariate assessment of clinical variables that potentially influence the S100 expression was done with a binary logistic regression. Female gender, convexity/falx and spinal meningioma location as well as NF2 and WHO grade 1 were associated with higher immunopositivity rates for S100 expression. Patient age at diagnosis, MIB1 immunopositivity and recurrent tumor status did not show an independent association with S100 expression. Details of the nominal logistic regression are displayed in Table 2.

**Table 2:** Binary logistic regression of factors associated with S100 expression

	Odds ratio (95% CI)	*p* value (Prob > Chisq)
Female gender	2.08 (1.39–3.09)	0.0003*
Age	–	0.1715
Location		
Convexity/falx vs. skull base	3.40 (2.39–4.82)	< 0.0001*
Spinal vs. skull base	3.42 (2.12–5.49)	< 0.0001*
Spinal vs. convexity/falx	1.01 (0.64–1.58)	0.9804
Primary vs. recurrent meningioma	1.42 (0.78–2.60)	0.2562
NF2 vs. sporadic	4.62 (2.44–8.74)	< 0.0001*
CNS WHO grading		
1 vs. 2	1.82 (1.13–2.94)	0.0133*
1 vs. 3	–	0.9973
2 vs. 3	–	0.9974
MIB1	–	0.5954

Asterisks (*) mark statistically significant results, *CI* confidence interval, *CNS* central nervous system, *MIB1* proliferation marker, *NF2* neurofibromatosis type 2, *Prob* probability, *WHO* World Health Organization

### S100 and progression-free survival

Information on radiographic tumor recurrence or progression was available for 1469 cases (88.0%) with a mean follow-up of 38.3 months ranging from 1.1 to 195.6 months. Tumor recurrence/progression was observed in 314 cases (21.4%).

**Fig. 2:**
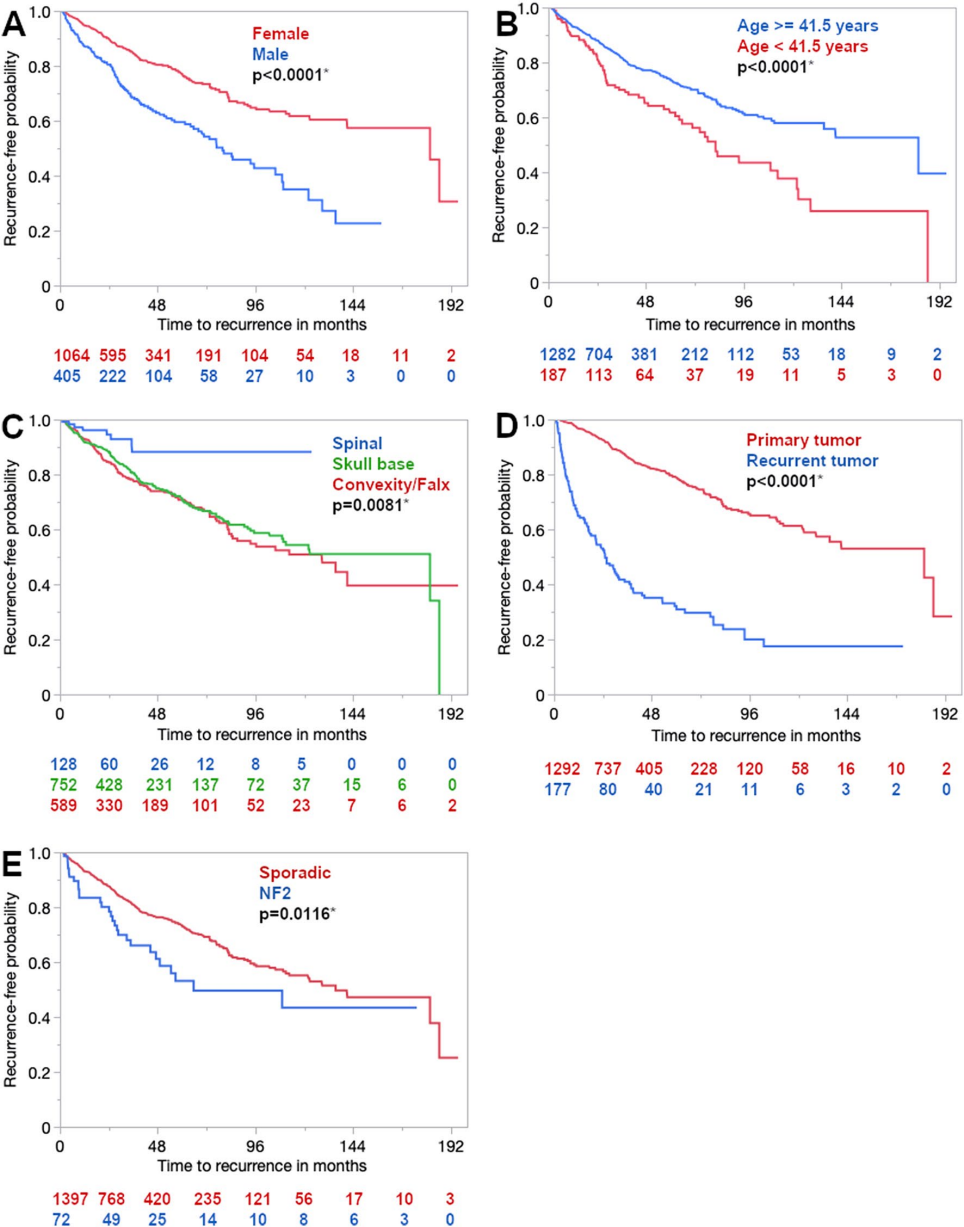
Kaplan–Meier curves for progression-free survival in the Tübingen Meningioma Cohort regarding gender (**A**), age (**B**), tumor location (**C**), primary/recurrent tumor status before surgery (**D**) and neurofibromatosis2/sporadic meningiomas (**E**). Asterisks (*) mark statistically significant differences

**Fig. 3:**
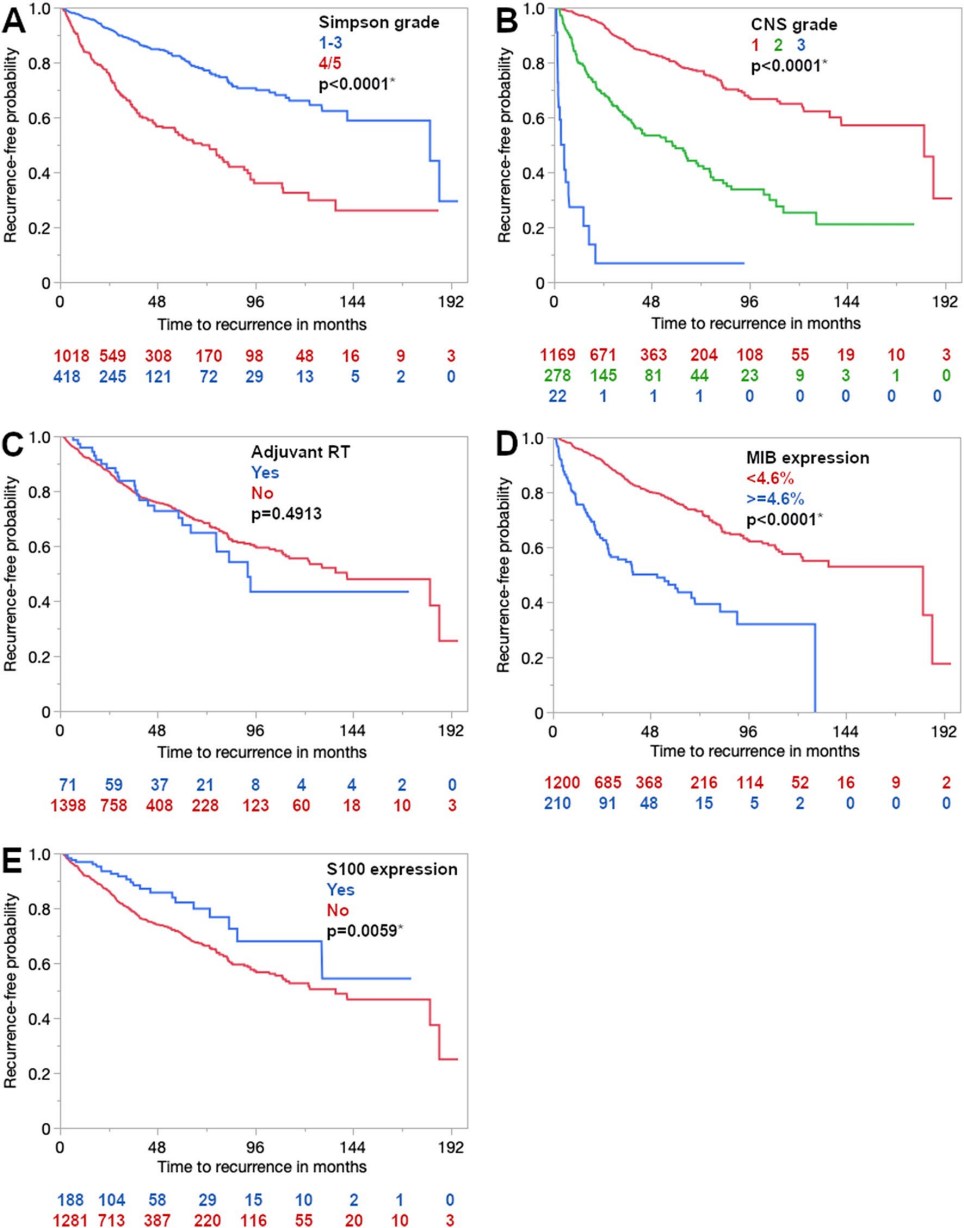
Kaplan–Meier curves for progression-free survival in the Tübingen Meningioma Cohort regarding extent of resection according to the Simpson grade (**A**), CNS grade according to the WHO classification (**B**), adjuvant radiotherapy (**C**), MIB1 expression (**D**) S100 expression (**E**). Asterisks (*) mark statistically significant differences

Male patients had a higher rate of tumor recurrence compared to female patients (32.4% vs. 17.2%, *p* < 0.0001). At the CART-specific age cutoff based on the maximum difference regarding tumor recurrence, patients younger than 41.5 years at diagnosis had a significantly higher rate of tumor recurrence (36.4% compared to 19.2%, *p* < 0.0001). Spinal meningiomas showed a significantly lower rate of tumor recurrence (6.3%) than meningiomas with convexity/falx or skull base location (23.8% and 22.1%, respectively, *p* < 0.0001). Recurrent meningiomas had a fourfold risk of another recurrence when compared with primary tumors (63.3% vs. 15.6%, *p* < 0.0001). A small subgroup of patients suffering from NF2 were included in this cohort (*n* = 72). Such meningiomas had a higher rate of recurrence (38.9% compared to 20.5%, *p* = 0.0002). A higher grade of tumor resection without residual meningioma tissue (Simpson grade 1–3) as well as a lower WHO grade were associated with a markedly lower tumor recurrence rate (each *p* < 0.0001). An expression of the proliferative marker MIB1 reaching 4.6% or beyond was associated with a higher rate of tumor recurrences (17.3% compared to 43.8%, *p* < 0.0001). Tumors with immunopositivity for S100 were found to have a lower risk of recurrence (12.2% compared to 22.7%, *p* = 0.0011). Applying the univariate Cox proportional hazard analysis, a favorable progression-free survival was observed for female gender, older age, primary tumors, extent of resection, lower CNS WHO grading and lower MIB1 expression. For histology subtypes within each WHO grade, there was only a significant difference between psammomatous and transitional meningiomas. Furthermore, patients receiving adjuvant radiotherapy after resection showed a favorable progression-free survival in the univariate analysis. The detailed results of the univariate analyses are shown in Table 3. In the Kaplan–Meier analysis, the univariate prognostic impact was confirmed for all examined factors except adjuvant radiotherapy, as seen in the respective Kaplan–Meier curves in Figs. 2 and 3.

**Table 3:** Univariate analysis of factors associated with tumor recurrence (Pearson’s Chi-squared test and Cox proportional hazard analysis)

	*N* (%)	Tumor recurrence (*N*, %)		Pearson’s	Cox proportional hazard	
		Yes	No	*p* value	HR (95% CI)	*p* value
Gender						
Female	1064 (72.4)	183 (17.2)	881 (82.8)	< 0.0001*	0.76 (0.59–0.99)	0.0383*
Male	405 (27.6)	131 (32.4)	274 (67.7)		1.32 (1.01–1.70)	
Age						
≥ 41.5	1282 (87.3)	246 (19.2)	1036 (80.8)	< 0.0001*	0.72 (0.52–1.00)	0.0489*
< 41.5	187 (12.7)	68 (36.4)	119 (63.6)		1.39 (1.00–1.93)	
Location						
Skull base	752 (51.2)	166 (22.1)	586 (77.9)	< 0.0001*		n/s
Convexity/falx	589 (40.1)	140 (23.8)	449 (76.2)			
Spinal	128 (8.7)	8 (6.3)	120 (93.8)			
Prim/Rec						
Primary	1292 (88.0)	202 (15.6)	1090 (84.4)		0.39 (0.28–0.55)	< 0.0001*
Recurrent	177 (12.1)	112 (63.3)	65 (36.7)	< 0.0001*	2.55 (1.80–3.60)	
NF2						
Yes	72 (4.9)	28 (38.9)	44 (61.1)	0.0002*		n/s
No	1397 (95.1)	286 (20.5)	1111 (79.5)			
Simpson grade						
1/2/3	1018 (70.9)	137 (13.5)	881 (86.5)	< 0.0001*	0.37 (0.28–0.48)	< 0.0001*
4/5	418 (29.1)	171 (40.9)	247 (59.1)		2.73 (2.10–3–54)	
CNS WHO grading						
1	1169 (79.6)	167 (14.3)	1002 (85.7)	< 0.0001*	CNS WHO grade 1 vs. 2	0.0186*
2	278 (18.9)	128 (46.0)	150 (54.0)		0.09 (0.01–0.67)	
3	22 (1.5)	19 (86.4)	3 (13.6)			
Histology						
1						
Angiomatous	29 (2.0)	2 (6.9)	27 (93.1)		Psammomatous vs. transitional	
Fibroblastic	112 (7.6)	11 (9.8)	101 (90.2)	< 0.0001*		
Lymphocyte rich	1 (0.1)	0 (–)	1 (100.0)		0.23 (0.05–0.99)	0.0488*
Meningothelial	729 (49.6)	118 (16.2)	611 (83.8)			
Metaplastic	20 (1.4)	0 (–)	20 (100.0)			
Microcystic	28 (1.9)	4 (14.3)	24 (85.7)			
Psammomatous	48 (3.3)	2 (4.2)	46 (95.8)			
Secretory	42 (2.9)	5 (11.9)	37 (88.1)			
Transitional	146 (9.9)	23 (15.8)	123 (84.3)			
NOS	15 (1.0)	3 (20.0)	12 (80.0)			
2						
Atypical	248 (16.9)	116 (46.8)	132 (53.2)			n/s
Chordoid	29 (2.0)	11 (37.9)	18 (62.1)			
Clear cell	0 (–)	0 (–)	0 (–)			
3						
Anaplastic	16 (1.1)	15 (93.8)	1 (6.3)			n/s
Papillary	0 (–)	0 (–)	0 (–)			
Rhabdoid	6 (0.4)	4 (66.7)	2 (33.3)			
Adjuvant RT						
Yes	71 (4.8)	25 (35.2)	46 (64.8)	0.0036*	0.37 (0.24–0.58)	
No	1398 (95.2)	289 (20.7)	1109 (79.3)		2.68 (1.71–4.19)	< 0.0001*
MIB1						
< = 4.6% or missing	1200 (85.1)	208 (17.3)	992 (82.7)	< 0.0001*	0.49 (0.36–0.66)	
> 4.6%	210 (14.9)	92 (43.8)	118 (56.2)		2.03 (1.51–2.74)	< 0.0001*
S100 expression						
Yes	188 (12.8)	23 (12.2)	165 (87.8)	0.0011*		
No	1281 (87.2)	291 (22.7)	990 (77.3)			n/s

Asterisks (*) mark statistically significant results, *CI* confidence interval, *CNS* central nervous system, *MIB1* proliferation marker, *NOS* not otherwise specified, *NF2* neurofibromatosis type 2, *n/s* not significant, *WHO* World Health Organization, *RT* radiotherapy

### Multivariate Cox proportional hazard of progression-free survival

For the multivariate analysis of meningioma recurrence/progression, a Cox proportional hazard model was applied. Female gender remained an independent protective factor (*p* = 0.015) while patient age of 41.5 years or older merely showed a prognostically positive trend without statistical significance (*p* = 0.0627). Meningioma location and NF2 also missed statistical significance regarding progression-free survival. Primary tumor status and lower WHO grade were independent positive prognostic factors leading to a favorable progression-free survival (each *p* < 0.0001). Resection of all visible tumor tissue (Simpson 1–3) and adjuvant radiotherapy were treatment modalities that also showed and independent positive prognostic effect (each *p* < 0.0001). Expression of the proliferation marker MIB1 exceeding 4.6% was associated with a twofold risk of tumor recurrence (*p* < 0.0001) while S100 immunopositivity had no independent influence on progression-free survival (*p* = 0.6140). Details of the Cox proportional hazard analysis can be seen in Table 4.

**Table 4:** Multivariate analysis of prognostic factors of tumor recurrence (Cox proportional hazard)

	Hazard ratio (95% CI)	*p *value (Prob > Chisq)
Female Gender	0.73 (0.56–0.94)	0.0150*
Age > = 41.5	0.73 (0.53–1.02)	0.0627
Location		
Convexity/falx vs. skull base	0.80 (0.62–1.03)	0.0853
Convexity/falx vs. spinal	1.44 (0.66–3.12)	0.3565
Skull base vs. spinal	1.80 (0.84–3.88)	0.1312
Recurrent vs. primary meningioma	3.46 (2.61–4.59)	< 0.0001*
NF2 vs. sporadic	0.84 (0.51–1.41)	0.5182
Simpson grade 1–3 vs. 4/5	0.34 (0.26–0.44)	< 0.0001*
CNS WHO grading		
1 vs. 2	0.34 (0.25–0.45)	< 0.0001*
1 vs. 3	0.06 (0.03–0.12)	< 0.0001*
2 vs. 3	0.19 (0.10–0.24)	< 0.0001*
Adjuvant RT	0.33 (0.21–0.50)	< 0.0001*
MIB1 > 4.6%	2.15 (1.61–2.86)	< 0.0001*
S100 immunopositivity	0.89 (0.56–1.41)	0.6140

Asterisks (*) mark statistically significant results, *CNS* central nervous system, *MIB1* proliferation marker, *NF2* neurofibromatosis type 2, *Prob* probability, *WHO* World Health Organization, *RT* radiotherapy

## Discussion

There is still an unmet need to further refine the prognostication of meningiomas. While the majority of patients can be cured by complete surgical excision, some patients experience tumor recurrence over time. One study with a long-term follow-up spanning 25 years even revealed a recurrence rate of over 40% (Pettersson-Segerlind et al. 2011). Although these specific data reflect the results of treatment strategies several decades ago, the bottom line is—meningiomas recur besides our best treatment efforts. But especially tumors with a higher tendency to recur or aggressive behavior need to be identified as early as possible, in order to allow for early radiation therapy(Goldbrunner et al. 2016) or novel targeted treatment approaches (Brastianos et al. 2019). Several findings during the last years have strengthened our ability to be more precise in this regard. The current WHO classification for meningiomas remains the most important mean to identify patients at risk (Louis et al. 2021), while several aberrations such as mutations of the promotor of the telomere reverse transcriptase (TERT) (Sahm et al. 2016), loss of the histone trimethylation H3K27me3 (Behling et al. 2020) and loss of the cyclin-dependent kinase inhibitor 2A/B (CDKN2A/B) (Sievers et al. 2020) are important adjuncts when it comes to refining risk stratification of meningioma patients. Results from molecular data on TERT and CDKN2A/B are now used to identify high-risk tumors (Maas et al. 2021).

In this regard, there is evidence that increased levels of the calcium-binding protein S100B (Donato 1999) may be of use to identify meningiomas with a better progression-free survival. This was suggested by a recent integrative molecular classification analysis in 121 meningiomas. Among four prognostically distinct molecular subgroups, meningiomas with the most favorable outcome showed exceptionally high levels of S100B levels in the proteome-analysis (Nassiri et al. 2021). Since S100 can be easily detected via immunohistochemical staining and is widely used in neuropathology practice to identify nerve sheath tumors, it is an ideal biomarker candidate. With over one thousand patients with high-quality clinical and follow-up data, we applied the Tübingen Meningioma Cohort to explore the clinical significance of this emerging marker with prognostic potential.

The univariate analysis confirmed the favorable prognostic impact of S100 immunopositivity in meningiomas with approximately half the recurrence rate compared to immunonegative cases (12.2 vs. 22.7%). This supports the initial observation made in the previous proteome analysis (Nassiri et al. 2021). However, in the Cox proportional hazard analysis, strong S100 expression did not show an independent impact on progression-free survival. When further exploring the distribution of S100-strong meningiomas, significant differences among many clinical factors were observed, including patient age and the expression of the proliferation marker MIB1. When integrating all clinical factors (excluding treatment-related factors) in a linear regression model we found that S100-strong meningiomas were independently associated with female gender, lower WHO grade, NF2 and non-skull base location. Especially, female gender and lower WHO grade are independent positive prognostic factors. Therefore, the favorable prognostic effect of increased S100 expression seen in the univariate analysis is likely caused by these confounding factors.

Nonetheless, it is of interest why such differences of S100 expression exist. Although S100 expression is consistently seen in nerve sheath tumors and usually not associated with meningeal tumors, it is known that a small subset of meningiomas may also express S100. Early reports in the child age of immunohistochemistry reported up to 8% S100-positive tumors, which is similar to our observation rate of 13% (Meis et al. 1986). In contrast, a recent study reported up to 34% of meningiomas to be positive for S100; however, in this study, tumors were rated as S100 positive when at least 5% of tumor cells expressed S100 (Boulagnon-Rombi et al. 2017). The distribution and prognostic role of S100, however, has not been explored in detail. Hancq et al. observed significantly higher S100B scores in benign compared to atypical meningiomas (Hancq et al. 2004). Similar observations have been reported in non-small cell lung cancer, where high mRNA expression level of S100B was associated with better OS in NSCLC patients (Liu et al. 2018).

The main limitation of this study is its retrospective single-center design. A large consecutive cohort of surgically treated meningiomas was analyzed, thus meningiomas with little or no growth that are rather treated conservatively are underrepresented. Furthermore, the analyzed tissue samples (2 biopsy cylinders of 1 mm in diameter each) may not be suitable for immunohistochemical analysis. However, S100 shows quite homogenous staining and is, therefore, suitable for the applied methodology. The assessment was done by two researchers independently with an exceptionally high interobserver agreement. This underlines how well the routine use of this marker could be implemented.

## Conclusions

In conclusion, our results of a large meningioma dataset indicate that the positive prognostic impact of S100 is mainly attributable to confounding clinical factors. Further studies are warranted to explore the S100 distribution pattern in meningioma.
